# Neighborhood environmental factors associated with leisure walking in adolescents

**DOI:** 10.11606/s1518-8787.2020054002222

**Published:** 2020-05-28

**Authors:** Arieli Fernandes Dias, Anelise Reis Gaya, Maria Paula Santos, Caroline Brand, Andreia Nogueira Pizarro, Camila Felin Fochesatto, Thiago Monteiro Mendes, Jorge Mota, Adroaldo Cezar Araujo Gaya

**Affiliations:** I Universidade Federal do Rio Grande do Sul Escola de Educação Física, Fisioterapia e Dança Programa de Pós-Graduação em Ciências do Movimento Humano Porto AlegreRS Brasil Universidade Federal do Rio Grande do Sul. Escola de Educação Física, Fisioterapia e Dança. Programa de Pós-Graduação em Ciências do Movimento Humano. Grupo de pesquisa Projeto Esporte Brasil. Porto Alegre, RS, Brasil; II Universidade do Porto Faculdade de Desporto Centro de Investigação em Atividade Física, Saúde e Lazer Porto Portugal Universidade do Porto. Faculdade de Desporto. Centro de Investigação em Atividade Física, Saúde e Lazer. Porto, Portugal; III Universidade do Porto Centro de Estudos em Geografia e Ordenamento do Território Porto Portugal Universidade do Porto. Centro de Estudos em Geografia e Ordenamento do Território. Porto, Portugal

**Keywords:** Adolescent, Walking, Socioeconomic Factors, Built Environment, Environmental Health, Cross-Sectional Studies

## Abstract

**OBJECTIVE:**

To verify the associations of leisure walking with perceived and objective measures of neighborhood environmental factors stratified by gender and socioeconomic status (SES) in Brazilian adolescents.

**METHODS:**

Cross-sectional study with a random sample of 1,130 high school students (47.3% girls; aged 14 to 20 years old) from Porto Alegre, Brazil. Leisure walking and SES were self-reported by the adolescents. Perceived environmental factors were assessed through Neighborhood Environment Walkability Scale for Youth (NEWS-Y). Objective measures were evaluated using Geographic Information Systems, with road network calculated around the adolescent’s residential address, using 0.5km and 1.0km buffers. Data collection was carried out in 2017 and generalized linear regression models were used.

**RESULTS:**

Leisure walking was positively associated with access to services (0.5 km buffers [Odds ratio (OR) = 2.22] 1.0 km buffers [OR = 2.17]) and lower distance to parks and squares (0.5 km [OR=2.80] 1.0 km [OR = 2.73]) in girls from low SES. Residential density (0.5 km [OR = 1.57] 1.0 km [OR = 1.54]) and walkability index (0.5 km [OR = 1.17] 1.0 km [OR = 1.20]) were associated with leisure walking in girls from middle SES. Boys from low SES showed an inverse association between crime safety and leisure walking (0.5 km [OR = 0.59] 1.0 km [OR = 0.63]). Neighborhood recreation facilities was positively associated with leisure walking in middle SES (0.5 km [OR = 1.55] 1.0 km [OR = 1.60]). Land use mix (0.5 km [OR = 1.81] 1.0 km [OR = 1.81]), neighborhood recreation facilities (0.5 km [OR = 2.32] 1.0 km [OR = 2.28]) and places for walking (0.5 km [OR=2.07] 1.0 km [OR=2.22]) were positively associated with leisure walking in high SES.

**CONCLUSION:**

Environmental factors (objectively and subjectively measured) and leisure walking show association in boys and girls of different SES.

## INTRODUCTION

All health domains recognize physical activity as beneficial. A study developed in 17 countries, including Brazil, showed that higher physical activity levels lower the risk of mortality and cardiovascular disease in individuals from low, middle, and high-income countries^1^. Additionally, participate in physical activity, especially during leisure time, lowers the risk of incident coronary heart disease among young women^2^. However, the prevalence of leisure-time physical inactivity (lower than 60 min/day) was 54.3% in Brazilians adolescents; more than a quarter of adolescents (26.5%) reported no engagement in physical activity during leisure time^3^.

A systematic review by Bauman et al.^4^ showed that physical activity in low and middle-income countries is associated with demographic, biological, psychosocial, environmental, social, and cultural variables. In recent years, researchers have shown increased interest in the association between environmental correlates and physical activity. Regarding the young population and the specific domain of leisure physical activity, environmental characteristics such as distance to local facilities and home environment were negatively associated in Portuguese adolescents^5^. In Nigeria, residential density and availability/quality of infrastructures were positively associated with leisure-time moderate to vigorous physical activity (MVPA) in adolescents^6^.

In Brazil, evidences available from Curitiba^7,8^, São Paulo^9^ and Recife^10^ concerned adults, while few studies focused on youngsters. Adolescents from northeastern Brazil reported that “seeing other adolescents engaged in physical activities” and “seeing interesting things while walking” prompted recreational activities^11^. In the south, living near the beach increased leisure-time moderate and vigorous physical activity (MVPA) for adolescents^12^. Lima et al.^13^ identified that distance from home and number of recreational facilities in the neighborhood were associated with physical activity among adolescents, and that these relationships differ according to gender.

Literature about this topic in Brazilian youth is scarce. Therefore, our study adds new information to the field including both objective and perceived measures of the environment for each gender and different socioeconomic status (SES). Also, we explore data from a city without evidence on those aspects. Additionally, in developing countries, information about the built environment by SES can contribute to the development of public policies, increase physical activity and result in a healthier population. Thus, the aim of this study was to verify the associations between leisure walking and perceived and objective measures of neighborhood environmental factors stratified by SES and gender in Brazilian adolescents.

## METHODS

### Study Characteristics and Ethical Aspects

Cross-sectional and quantitative study developed in Porto Alegre, Brazil. The city has a population of approximately 1.4 million inhabitants (year 2010), a territorial area of 496.681 km^2^ and a demographic density of 2.837,53 inhabitants/km^214^. The study was approved by the Ethics Committee of Research with Human Beings of the *Universidade Federal do Rio Grande do Sul* (nº. 1,338.597).

### Participant Recruitment

The study sample comprised approximately 34,645 high school students, from 71 public schools^15^, allocated in the following regions: 8,057 north, 6,423 south, 4,268 east, and 15,897 central district.

To calculate the sample size, the following criteria were considered: a) estimated population of 34,645 students (N); b) proportion of 50% (p); c) complementary percentage of 100 – p (q); d) degree of confidence of 2 standard deviations (S); and e) acceptable sampling error of 3% (e). After adopting these criteria, the formula [n=S^2^.p.q.N/e^2^(N – 1) + (S^2^.p.q)] estimated that 1,077 students should be evaluated, as a representative sample of the population. However, to avoid difficulties with sample loss, an increase of 5% was assumed, totaling 1,130 adolescents. The power of the test was then calculated in the software G*power version 3.1.9.2, considering generalized linear regression, small effect size (F^2^ = 0.03), α < 0.05 and 19 predictors. Thus, the power of the test was 0.97.

Sample selection considered the proportion of youth enrolled in the schools by region; thus comprising: 263 students from 4 schools in the north region (23.26%); 518 students from 7 schools in the central region (45.88%); 140 students from 2 schools in the east region (12.32%); and 209 students from 3 schools in the south region (18.54%).

Sample selection was performed in multiple phases^16^. First, schools were selected according to each region, and then, in the schools, high school classes were randomly selected. A number was assigned for each school and all numbers were placed in a box, mixed and randomly reelected one by one. Then, data was collected in one class from each high school year. All students from each class selected were invited to participate in the study. Inclusion criteria were: a) belong to the first, second or third year of high school; b) hand in the informed consent form signed by a parent or guardian; and c) sign the assent form. According to Sawyer et al.^17^ 10–24 years range corresponds more closely to adolescent growth and popular understandings of this life phase; thus, we use the term adolescents, even when some students were over 18 years of age.

### Data Collection

Data was collected during an eight-month period in 2017. First, the researcher went to the selected schools, explained the study and if the principals agreed to participate, they were asked to sign an acceptance form. Then data collection was scheduled. Questionnaires were filled out during a regular class, corresponding to approximately 45 minutes and data confidentiality was kept.

### Measures

#### Leisure physical activity

Self-reported leisure physical activity was assessed by their respective sections of the long form of the International Physical Activity Questionnaire (IPAQ)^18^. This instrument was translated and adapted culturally in several countries^18^, including Brazil, and has been applied in epidemiological studies in Latin American^19^. To assess leisure walking, subjects answered the question ‘How many days per week do you usually walk in your free time?’. Response choices ranged from zero to seven. We created one binary outcome: participation in leisure-time walking (or not).

#### Perceived environment

To measure perceived neighborhood environmental factors, the version of the Neighborhood Environment Walkability Scale for Youth (NEWS-Y)^20^ validated in Brazil^21^ was used. This questionnaire evaluates perceived environmental factors that may influence youth physical activity^20^. Questions were considered according to the following dimensions, proposed by the NEWS-Y scoring guidelines^22^: Land use mix-diversity (perception of distance from home to a variety of common destinations, such as shops or school), neighborhood recreation facilities (perceived distance from the student house, walking to a variety of places for physical activity practice, such as walking/running track or large public park), access to services, street connectivity, places for walking, neighborhood aesthetics, neighborhood safety and crime safety. More information can be found in Lima et al.^21^, Rosenberg et al.^20^ and through the link <http://sallis.ucsd.edu/Documents/Measures_documents/NEWS_Y_adolescent.pdf>.

For land use mix-diversity and neighborhood recreation facilities, the answer options were: 1–5min, 6–10min, 11–20min, 21–30min, more than 30 min and don’t know/there isn’t. The option ‘don’t know’ was coded as “more than 30 min” because if the facility is not within walking distance the actual walk is likely more than 31 minutes^22^. All items were reverse coded and employed mean values.

All the questions from the dimensions access to services, street connectivity, places for walking, neighborhood aesthetics, neighborhood and crime safety were measured using 4-point Likert scale (strongly disagree, partially disagree, partially agree, strongly agree). All determinants were calculated following the NEWS-Y scoring guidelines^22^, with a higher score indicating better conditions for physical activity.

#### Objective environment factors

From the adolescent’s addresses reported in the questionnaire, we performed a georeferencing process and represented them in the Geographic System Information (GIS) environment through ArcMap 10.3.1 software. The shapefile of the streets, parks and squares provided by the Municipal Department of Urbanism, Environment and Sustainability of Porto Alegre – RS were used for the analyses.

The distances between houses and parks or squares of the city were defined by the tool “Network Analyst/Closest Facility.” The distance was calculated in meters and categorized in tertiles of “close,” “medium” and “far.” Buffers within 0.5km and 1.0km of the participants’ homes, reachable by the street network, were defined to estimate accessible neighborhood features. Thus, the following variables were used: existence of parks and squares (existence of parks and squares in buffer); existence of bicycle path (existence of bicycle path in buffer); residential density (number of residences within each buffer); density of blocks (number of blocks within each buffer); average size of the blocks (average size of streets/blocks within each buffer); connectivity between streets (intersection number of streets in buffer); and walkability index (sum 2* z-score connectivity between streets + z-score residential density).

#### Socioeconomic status

SES was assessed through a questionnaire that included the number of owned items at the adolescents’ residence and the level of schooling of the parent or guardian. By means of the criteria established by the Brazilian Market Research Association^23^, a score was assigned to each answer, and the sum of the points identified each student’s economic class^23^. Then adolescents were classified into low (1° tertile), middle (2° tertile) and high (3° tertile) class.

## Data Analyses

Descriptive data are shown as absolute and relative values, means and standard deviations stratified by gender and SES. Geographical data of the squares, parks, bicycle paths and students’ homes are presented in the figure.

**Figure:**
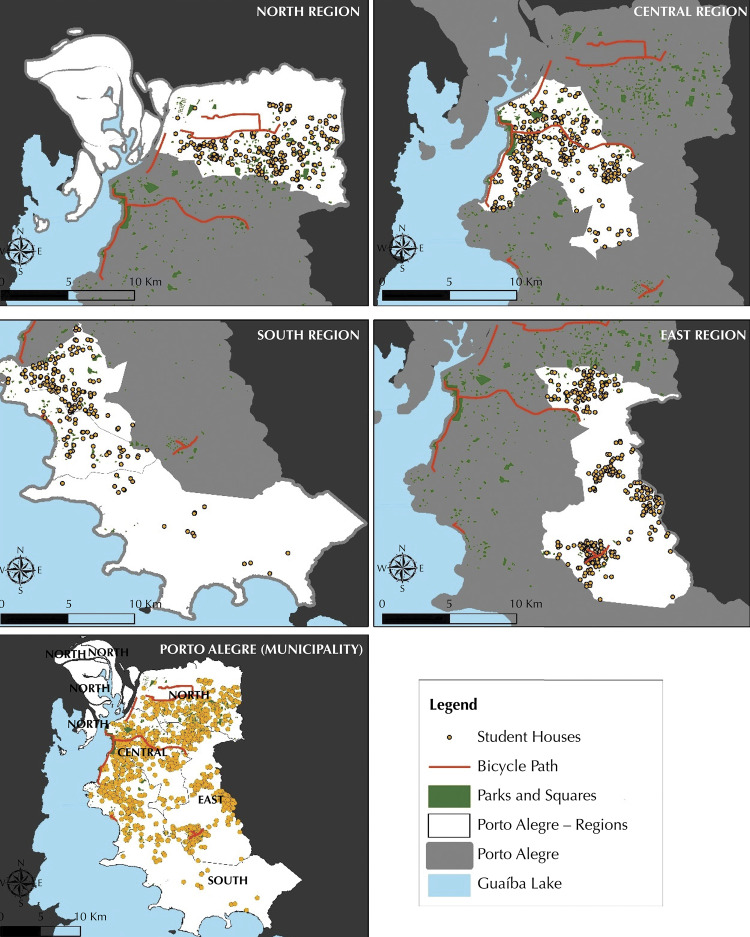
Georeferencing of students’ residence, parks, squares, and bicycle path in Porto Alegre (RS), Brazil, 2017 (n = 1010).

The internal consistency from NEWS-Y dimensions variables was verified through Cronbach Alpha (0.89), indicating 0.89 as an acceptable reliability. Values for each dimension were: land use mix-diversity (0.88), neighborhood recreation facilities (0.84), access to services (0.36), street connectivity (0.36), places for walking (0.30), neighborhood aesthetics (0.71), neighborhood safety (0.10) and crime safety (0.85).

Generalized linear regressions were used to test the association between perceived and objective measures of neighborhood environmental factors with leisure walking. Thus, the analyses were splitted by gender and SES into two models: variables of the perceived environment adding objective built environment with 0.5 kilometers buffers (model 1); and perceived environment adding objective built environment with 1.0 kilometer buffers (model 2). All analyses were adjusted for age and region and we tested additional adjustment for the environmental variables considering collinearity between them (rho ≥ 0.60). Variables with high collinearity were described in the legend of the tables. All analyses were carried out using the IBM SPSS 22 (SPSS, Inc., Chicago, Illinois, USA), α < 0.05 was adopted and confidence intervals (95%) were presented.

## RESULTS

Although the study sample comprised 1,130 adolescents from Porto Alegre-RS, the SES data refers only to 1,113 students. Sample loss occurred because the adolescents responded the questionnaire inadequately.

Table 1 shows the descriptive characteristics of the sample, as well as data concerning perceived and objective measures of neighborhood environmental factors by sex and SES. We observed a higher prevalence of adolescents that engage in leisure walking in boys and girls from high SES.

**Table 1:** Descriptive characteristics of the sample stratified by gender and socioeconomic status in adolescents from Porto Alegre (RS), Brazil, 2017.

Variables	Girls			Boys		
	
	SES (n = 587)			SES (n = 526)		
	
	Low (n = 189)	Middle (n = 201)	High (n = 197)	Low (n = 175)	Middle (n = 180)	High (n = 171)
					
	n (%)	n (%)	n (%)	n (%)	n (%)	n (%)
**Age**						
14–15	32 (16.9)	35 (17.4)	46 (23.4)	23 (13.1)	25 (13.9)	44 (25.7)
16–17	129 (68.3)	136 (67.7)	137 (69.5)	117 (66.9)	118 (65.6)	105 (61.4)
18–20	28 (14.8)	30 (14.9)	14 (7.1)	35 (20.0)	37 (20.6)	22 (12.9)
**Region**						
Central	88 (46.6)	100 (49.8)	103 (52.3)	58 (33.1)	70 (38.9)	92 (53.8)
North	33 (17.5)	48 (23.9)	48 (24.4)	42 (24.0)	48 (26.7)	28 (16.4)
South	36 (19.0)	33 (16.4)	32 (16.2)	38 (21.7)	39 (21.7)	39 (22.8)
East	32 (16.9)	20 (10.0)	14 (7.1)	37 (21.1)	23 (12.8)	12 (7.0)
**Leisure PA**						
Leisure Walking (proportion: yes)	97 (51.3)	94 (46.8)	107 (54.3)	103 (58.9)	102 (56.7)	111 (64.9)
**Objective (GIS)**						
Existence of parks and squares (0.5km)	81 (48.2)	109 (59.9)	109 (61.6)	98 (62.0)	103 (63.6)	103 (67.8)
Existence of parks and squares (1.0km)	132 (78.6)	160 (87.9)	156 (88.1)	134 (84.8)	136 (84.0)	141 (92.8)
Existence of bicycle path (0.5km)	12 (7.1)	16 (8.8)	27 (15.3)	26 (16.5)	24 (14.8)	15 (9.9)
Existence of bicycle path (1.0km)	30 (17.9)	44 (24.2)	50 (28.2)	43 (27.2)	37 (22.8)	35 (23.0)

	**Mean (SD)**	**Mean (SD)**	**Mean (SD)**	**Mean (SD)**	**Mean (SD)**	**Mean (SD)**

**Perceived Environment**						
Land use mix – diversity	3.16 (0.85)	3.54 (0.89)	3.73 (0.84)	3.45 (0.81)	3.66 (0.81)	3.83 (0.94)
Neighborhood recreation facilities	2.25 (0.87)	2.73 (1.05)	2.97 (0.96)	2.82 (0.99)	3.03 (1.04)	3.49 (1.00)
Access to services	2.81 (0.51)	2.97 (0.51)	3.01 (0.48)	2.98 (0.48)	2.93 (0.47)	3.05 (0.45)
Street connectivity	2.68 (0.68)	2.69 (0.70)	2.79 (0.67)	2.75 (0.68)	2.76 (0.67)	2.81 (0.66)
Places for walking	2.54 (0.80)	2.79 (0.69)	2.77 (0.66	2.81 (0.74)	2.75 (0.59)	2.87 (0.61)
Neighborhood aesthetics	2.30 (0.73)	2.50 (0.69)	2.70 (0.67)	2.55 (0.73)	2.56 (0.71)	2.76 (0.69)
Neighborhood safety	2.56 (0.48)	2.44 (0.44)	2.37 (0.47)	2.28 (0.42)	2.32 (0.41)	2.29 (0.47)
Crime safety	2.86 (0.82)	2.76 (0.80)	2.59 (0.76)	2.38 (0.79)	2.46 (0.73)	2.36 (0.78)
**Objective (GIS)**						
Lower distance for parks and squares	818.90 (1,089.89)	558.43 (611.24)	611.98 (999.72)	649.18 (1,055.49)	696.78 (1,246.78)	453.92 (457.31)
Lower distance for bicycle path	3,026.72 (2,094.62)	2,507.85 (1,757.05)	2,285.94 (1,913.92)	2,607.55 (1,986.55)	2,628.14 (2,015.07)	2,359.63 (1,621.13)
**Objective Environment Factors (0.5 km-buffers)**						
Residential density	2,550.35 (1,254.49)	2,855.59 (1,456.52)	2,811.36 (1,540.46)	2,728.11 (1,461.43)	2,583.06 (1,373.02)	3,003.98 (1,578.16)
Connectivity between streets	53.61 (33.53)	51.94 (31.46)	47.20 (27.46)	50.83 (33.42)	49.84 (33.10)	50.11 (27.92)
Blocks density	7,973.63 (3,469.49)	8,765.06 (3,516.94)	8,780.96 (3,409.79)	8,627.06 (3,893.09)	8,515.05 (3,774.66)	9,231.46 (3,279.48)
Average size of the blocks	120.15 (123.73)	121.61 (63.89)	134.17 (123.79)	118.99 (54.31)	118.76 (44.33)	122.75 (44.81)
Walkability index*	0.02 (2.69)	0.15 (2.54)	-0.19 (2.25)	-0.05 (2.64)	-0.17 (2.63)	0.15 (2.29)
**Objective Environment Factors (1.0 km-buffers)**						
Residential density	6,179.16 (3,488.29)	7,464.60 (4,053.18)	7,862.40 (4,562.06)	6,989.69 (4,136.42)	6,970.60 (3,727.70)	8,086.56 (4,368.71)
Connectivity between streets	180.61 (104.48)	193.36 (104.54)	178.90 (94.90)	185.15 (107.83)	187.33 (111.07)	186.56 (92.36)
Blocks density	25,397.55 (11,714.66)	29,766.52 (11,850.19)	30,480.68 (12,090.82)	28,926.19 (13,294.79)	29,464.51 (12,476.15)	30,918.12 (10,734.35)
Average size of the blocks	107.47 (40.74)	112.55 (44.83)	121.65 (43.56)	112.99 (39.70)	116.68 (39.55)	118.53 (39.69)
Walkability index*	-0.35 (2.62)	0.20 (2.58)	-0.01 (2.45)	-0.06 (2.68)	-0.07 (2.69)	0.22 (2.31)

*Standardized variables (transformed into z-scores).

The geocoding of residential addresses for built environmental analysis (objective measures) comprised 1,010 adolescents. Sample losses correspond to lack of address information and incompatibilities in the street network. The Figure presents the environmental characteristics, such as existence of bicycle path, parks, and squares, as well as georeferencing of students’ residence. We observe an unequal distribution of park and squares around the city, and few bicycle paths.

Table 2 and 3 show the results of perceived and observed neighborhood environmental factors and leisure walking by gender and SES. Girls in 0.5 and 1.0km buffers had similar results (Table 2. Model 1 and 2). The analysis for low SES adolescent girls indicated that access to services and lower distance for parks and squares were positively associated with walking leisure. The objective measures, such as residential density and walkability index, were associated with walking leisure in middle SES. Girls of high SES showed no associations.

**Table 2:** Association between perceived and objective neighborhood environmental factors and leisure walking by socioeconomic status in adolescent girls from Porto Alegre (RS), Brazil, 2017.

Perceived Environment	Leisure walking					

	Model 1^a^			Model 2^a^		
	
	SES (n = 587)			SES (n = 587)		
	
	Low (n = 189)	Middle (n = 201)	High (n = 197)	Low (n = 189)	Middle (n = 201)	High (n = 197)
					
	OR (CI-95%)	OR (CI-95%)	OR (CI-95%)	OR (CI-95%)	OR (CI-95%)	OR (CI-95%)
Land use mix – diversity	0.79 (0.48–1.33)	1.20 (0.81–1.76)^b^	1.18 (0.73–1.90)	0.79 (0.47–1.32)	1.07 (0.70–1.63)^b^	1.17 (0.71–1.93)
Neighborhood recreation facilities	0.76 (0.46–1.25)	1.33 (0.95–1.86)^b^	1.19 (0.79–1.79)	0.81 (0.50–1.32)	1.14 (0.81–1.60)^b^	1.09 (0.73–1.62)
Access to services	**2.22 (1.01**–**4.92)**	1.12 (0.58–2.16)	0.92 (0.42–2.02)	**2.17 (1.01**–**4.83)**	0.99 (0.50–1.97)	0.93 (0.42–2.05)
Street connectivity	0.72 (0.43–1.21)	1.13 (0.68–1.87)	1.27 (0.74–2.16)	0.69 (0.40–1.17)	1.02 (0.62–1.67)	1.41 (0.83–2.40)
Places for walking	0.74 (0.46–1.18)	0.80 (0.45–1.42)	1.03 (0.60–1.74)	0.80 (0.50–1.26)	0.87 (0.50–1.53)	1.01 (0.59–1.71)
Neighborhood aesthetics	1.09 (0.65–1.81)	1.30 (0.78–2.17)	1.51 (0.87–2.63)	1.12 (0.68–1.85)	1.20 (0.73–1.98)	1.38 (0.80–2.39)
Neighborhood safety	0.81 (0.35–1.84)	1.14 (0.53–2.45)	0.65 (0.29–1.46)	0.89 (0.39–1.99)	1.19 (0.56–2.55)	0.68 (0.30–1.54)
Crime safety	0.93 (0.61–1.41)	0.83 (0.54–1.28)	0.75 (0.47–1.20)	0.91 (0.59–1.38)	0.82 (0.54–1.27)	0.71 (0.44–1.15)
**Objective (GIS)**						
Lower distance for parks and squares						
1 tertile (close)	**2.80 (1.04**–**7.55)**^b^	0.56 (0.22–1.39)^b^	0.42 (0.15–1.16)^b^	**2.73 (1.01**–**7.32)**	0.47 (0.18–1.19)	0.55 (0.20–1.48)
2 tertile (medium)	2.35 (0.93–5.94)^b^	0.67 (0.30–1.52)^b^	0.44 (0.17–1.17)^b^	2.36 (0.91–6.14)	0.54 (0.23–1.27)	0.56 (0.21–1.47)
3 tertile (far)	1	1	1	1	1	1
Lower distance for bicycle path						
1 tertile (close)	1.88 (0.72–4.88)	2.09 (0.86–5.04)	0.51 (0.19–1.32)^b^	1.91 (0.68–5.36)^b^	2.14 (0.86–5.31)^b^	0.57 (0.21–1.50)^b^
2 tertile (medium)	1.37 (0.63–2.95)	1.93 (0.85–4.35)	0.48 (0.20–1.17)^b^	1.42 (0.58–3.42)^b^	1.75 (0.78–3.96)^b^	0.52 (0.21–1.30)^b^
3 tertile (far)	1	1	1	1	1	1
**Objective Environment Factors**	(0.5km - buffers)			(1km – buffers)		
Existence of parks and squares						
No	1	1	1	1	1	1
Yes	1.63 (0.75–3.53)^b^	0.74 (0.36–1.52)^b^	0.73 (0.34–1.58)^b^	1.04 (0.45–2.43)^b^	1.54 (0.49–4.80)	0.30 (0.07–1.17)
Existence of bicycle path						
No	1	1	1	1	1	1
Yes	2.16 (0.47–9.93)	1.80 (0.52–6.16)	0.77 (0.31–1.91)^b^	1.70 (0.68–4.26)^b^	0.99 (0.44–2.20)^b^	0.79 (0.37–1.69)^b^
Residential density*	0.99 (0.62–1.57)^b^	**1.57 (1.08**–**2.28)**^b^	1.21 (0.83–1.75)^b^	0.88 (0.51–1.51)^b^	**1.54 (1.05**–**2.25)**^b^	1.25 (0.84–1.84)^b^
Connectivity between streets*	0.92 (0.65–1.30)^b^	1.25 (0.89–1.76)^b^	1.01 (0.67–1.51)^b^	1.02 (0.72–1.43)^b^	1.14 (0.80–1.61)^b^	1.90 (0.64–1.45)^b^
Blocks density*	1.11 (0.77–1.61)^b^	1.16 (0.79–1.71)^b^	1.11 (0.74–1.58)^b^	1.00 (0.68–1.47)^b^	1.35 (0.92–1.98)^b^	1.13 (0.72–1.77)^b^
Average size of the blocks*	1.08 (0.83–1.42)^b^	0.90 (0.57–1.43)^b^	0.86 (0.58–1.29)^b^	1.12 (0.76–1.67)^b^	0.78 (0.51–1.19)^b^	0.77 (0.52–1.15)^b^
Walkability index*	0.93 (0.81–1.08)^b^	**1.17 (1.02**–**1.35)**^b^	1.09 (0.92–1.30)^b^	0.94 (0.80–1.09)^b^	**1.20 (1.04**–**1.39)**^b^	1.06 (0.89–1.26)^b^

*Standardized variables (transformed into z-scores). ^a^ Adjusted for age and region. ^b^ Adjusted for model 1^a^, without the environmental variables that present high collinearity (rho ≥ 0.60).

Regarding boys, only perceived environmental factors were associated with leisure walking in both models (Table 3). For boys from low SES showed an inverse association between crime safety and leisure walking. Neighborhood recreation facilities was positively associated with leisure walking in middle SES. Land use mix, neighborhood recreation facilities and places for walking were positively associated with leisure walking in high SES.

**Table 3:** Association between perceived and objective neighborhood environment factors and leisure walking by socioeconomic status in adolescent boys from Porto Alegre (RS), Brazil, 2017.

Perceived Environment	Leisure walking					

	Model 1^a^			Model 2^a^		
	
	SES (n = 526)			SES (n = 526)		
	
	Low (n = 175)	Middle (n = 180)	High (n = 171)	Low (n = 175)	Middle (n = 180)	High (n = 171)
					
	OR (CI-95%)	OR (CI-95%)	OR (CI-95%)	OR (CI-95%)	OR (CI-95%)	OR (CI-95%)
Land use mix – diversity	0.84 (0.47–1.51)	1.30 (0.81–2.07)^b^	**1.81 (1.09**–**3.02)**^b^	0.84 (0.47–1.51)	1.40 (0.91–2.18)^b^	**1.81 (1.11**–**2.94)**^b^
Neighborhood recreation facilities	1.45 (0.89–2.36)	**1.55 (1.06**–**2.29)**^b^	**2.32 (1.26**–**3.93)**^b^	1.38 (0.85–2.23)	**1.60 (1.09**–**2.35)**^b^	**2.28 (1.35**–**3.86)**^b^
Access to services	0.59 (0.24–1.45)	1.40 (0.64–3.02)	1.06 (0.75–1.51)	0.57 (0.23–1.40)	1.27 (0.57–2.79)	1.09 (0.77–1.55)
Street connectivity	1.01 (0.60–1.71)	0.84 (0.50–1.43)	0.57 (0.30–1.08)	1.04 (0.61–1.78)	0.94 (0.55–1.63)	0.56 (0.30–1.04)
Places for walking	0.94 (0.56–1.57)	1.07 (0.57–2.00)	**2.07 (1.03**–**4.19)**	1.01 (0.60–1.69)	0.99 (0.53–1.86)	**2.22 (1.10**–**4.46)**
Neighborhood aesthetics	1.15 (0.68–1.96)	1.01 (0.60–1.70)	1.54 (0.85–2.78)	1.05 (0.62–1.77)	0.89 (0.52–1.53)	1.47 (0.82–2.63)
Neighborhood safety	1.59 (0.58–4.38)	1.31 (0.56–3.06)	1.15 (0.46–2.90)	1.44 (0.53–3.91)	1.36 (0.58–3.17)	1.34 (0.53–3.38)
Crime safety	**0.59 (0.37**–**0.95)**	0.92 (0.58–1.48)	0.93 (0.55–1.56)	**0.63 (0.36**–**0.98)**	0.89 (0.55–1.45)	0.87 (0.53–1.42)
**Objective (GIS)**						
Lower distance for parks and squares						
1 tertile (close)	0.42 (0.15–1.14)^b^	0.33 (0.11–1.01)^b^	0.40 (0.15–1.04)^b^	0.37 (0.13–1.01)	0.35 (0.11–1.02)	0.51 (0.18–1.46)
2 tertile (medium)	0.47 (0.18–1.23)^b^	0.67 (0.27–1.66)^b^	0.74 (0.26–2.09)^b^	0.40 (0.14–1.11)	0.67 (0.23–1.67)	0.91 (0.31–2.66)
3 tertile (far)	1	1	1	1	1	1
Lower distance for bicycle path						
1 tertile (close)	0.86 (0.33–2.24)	1.13 (0.36–3.51)	0.51 (0.14–1.80)	0.89 (0.33–2.41)^b^	1.61 (0.53–4.87)^b^	0.50 (0.15–1.66)^b^
2 tertile (medium)	1.93 (0.75–4.98)	0.78 (0.32–1.87)	0.35 (0.10–1.21)	1.83 (0.71–4.74)^b^	0.77 (0.30–1.93)^b^	0.43 (0.12–1.48)^b^
3 tertile (far)	1	1	1	1	1	1
**Objective Environment Factors**	(0.5km - buffers)			(1km – buffers)		
Existence of parks and squares						
No	1	1	1	1	1	1
Yes	1.46 (0.60–3.53)^b^	0.80 (0.36–1.77)^b^	0.49 (0.20–1.17)^b^	2.77 (0.74–10.30)	0.91 (0.28–2.97)	0.96 (0.20–4.50)
Existence of bicycle path						
No	1	1	1	1	1	1
Yes	0.79 (0.57–1.09)	2.70 (0.93–7.85)	0.35 (0.10–1.22)	2.30 (0.98–5.25)^b^	1.73 (0.71–4.24)^b^	0.43 (0.15–1.18)^b^
Residential density*	1.21 (0.79–1.85)^b^	0.80 (0.55–1.17)^b^	1.07 (0.70–1.64)^b^	1.31 (0.86–1.99)^b^	0.70 (0.43–1.16)^b^	1.16 (0.74–1.83)^b^
Connectivity between streets*	1.27 (0.84–1.94)^b^	1.05 (0.73–1.50)^b^	1.03 (0.61–1.74)^b^	1.01 (0.66–1.56)^b^	1.10 (0.75–1.61)^b^	0.61 (0.36–1.04)^b^
Blocks density*	1.56 (0.96–2.53)^b^	0.69 (0.47–1.02)^b^	1.31 (0.77–2.25)^b^	1.37 (0.86–2.18)^b^	0.76 (0.49–1.16)^b^	0.97 (0.56–1.68)^b^
Average size of the blocks*	0.90 (0.50–1.61)^b^	1.12 (0.56–2.25)^b^	1.23 (0.56–2.69)^b^	1.21 (0.75–1.98)^b^	0.96 (0.64–1.45)^b^	1.46 (0.87–2.43)^b^
Walkability index*	1.09 (0.93–1.28)^b^	1.02 (0.88–1.17)^b^	0.97 (0.81–1.17)^b^	1.13 (0.96–1.33)^b^	1.01 (0.88–1.17)^b^	0.87 (0.72–1.04)^b^

*Standardized variables (transformed into z-scores). ^a^ Adjusted for age and region. ^b^ Adjusted for model 1^a^, without the environmental variables that present high collinearity (rho ≥ 0.60).

## DISCUSSION

The main findings of this study show an association between leisure walking and perceived and objective measures of neighbourhood environmental factors, regarding gender and SES in adolescents.

For girls, results indicated that access to services and lower distance to parks and squares were positively associated with leisure walking in low SES. Studies in Argentina^24^ and Brazil^13^ present similar results, adjusting for SES. Additionally, Pereira et al.^5^ analyses disregarded SES, which may have influenced the results as evidence shows that SES affect the relationship between environmental factors and leisure physical activity.

Our study also showed that residential density and walkability index are associated with leisure walking in middle SES girls. Some studies have indicated association between walkability and physical activity in adolescents, but in low and high SES^25,26^. One explanation for this discrepancy can be that these studies considered neighbourhood SES levels instead of individual ones. Additionally, residential density was positively related to walking in adolescents when adjusted for SES.

Results of this study expand on the current knowledge about associations of leisure walking with both perceived and objective measures of neighbourhood environment in adolescents, when considering the impact SES and gender have in leisure physical activity. Most of the evidence on this topic originated from developed countries, where SES may represent a non-intervenient factor.

The lack of association between environmental factors and leisure walking in high SES was unexpected. With no apparent reason for this outcome, we hypothesize that variables unaddressed in this study, such as social support or owning a car, may be influencing this result. Furthermore, adolescents of high SES may have more access to training courses and gyms, which could decrease the time availability for leisure walking. Among girls, a shorter distance to squares and parks, a favourable perception of access in the neighborhood as well as high walkability index and residential density are intervenient factors for leisure walking, and these results varied according to SES.

Among boys, crime safety was inversely associated with leisure walking in low SES. We expected to find similar association in middle and high SES, considering the high crime rates in Brazil and that perceived lack of safety constrains physical activity behaviours^27^. Agreeing with our result, Mitáš et al.^28^ indicated that boys who met recommendations for leisure-time walking were the ones who perceived the safest neighborhood environment. In middle and high SES, our results showed that neighborhood recreation facilities were associated with leisure walking, besides land use mix and places for walking. Even though some studies point to the association between shorter distance to parks and number of facilities near home with physical activity in boys^13,29^, literature lacks evidence regarding the influence of SES in this relationship Additionally, systematic reviews have shown that access and neighborhood park environment characteristics was associated with physical activity^30^. Therefore, data concerning different SES indicated that perceived environmental factors, such as crime safety, land use mix, neighborhood recreation facilities and places for walking are important factors for leisure walking in adolescent boys.

We encountered some limitations when interpreting results. The cross-sectional design does not allow for cause and effect to be determined. Additionally, some characteristics that can be associated with the outcome were not assessed, such as motivation and social support. The walkability index did not consider land use mix and retail floor area ratio. Finally, leisure walking was self-reported.

Strengths of this study are the large sample size and using both perceived and objective measures of the environmental factors. Additionally, to our knowledge this was one of the first studies to account for SES and gender in the association between environmental factors and leisure walking in Brazilian adolescents. Considering these aspects, our findings can help public administrations develop strategies to promote physical activity, since some environment characteristics may be important tools to increase healthy behaviour in the population. Also, our results serve to understand how these relationships take place in each gender and SES, thereby contributing to the development of targeted actions for each context. Future studies should use longitudinal designs to analyse the association between environmental factors and leisure walking over time. Another issue is to understand the reasons and other social factors that lead adolescents to leisure walking.

In conclusion, we found an association between environmental factors (objectively and subjectively measured) and leisure walking in boys and girls of different SES. The influence of gender and SES must be considered when approaching environment and physical activity in Brazilian adolescents.

Low (0.5km buffers) – High correlation between following variables: Residential density with blocks density (rho = 0.72), with connectivity between streets (rho = 0.66) and walkability (rho = 0.81); Blocks density with connectivity between streets (rho = 0.86), lower distance for park and squares (rho = -0.61), existence of park and square (rho = 0.60) and walkability (rho = 0.89). Connectivity between streets with average size of the blocks (rho = -0.80) and walkability (rho = 0.95). Average size of the blocks with walkability (rho = -0.73). Existence of park and square with lower distance for park and square (rho = -0.84).

Low (1km buffers) – High correlation between following variables: Residential density with connectivity between streets (rho = 0.77), blocks density (rho = 0.85), existence of park and square (rho = 0.60) and walkability (rho = 0.87). Connectivity between streets with blocks density (rho = 0.90), average size of the blocks (rho = -0.69), existence of park and square (rho = 0.61) and walkability (rho = 0.96). Blocks density with walkability (rho = 0.93) and existence of park and square (rho = 0.63). Walkability with average size of the blocks (rho = -0.61) and existence of park and square (rho = 0.62). Lower distance for bicycle path with existence of bicycle path (rho = -0.64).

Middle (0.5km buffers) – High correlation between following variables: Land use mix with neighborhood recreation facilities (rho = 0.61); residential density with blocks density (rho = 0.62) and walkability (rho = 0.76). Blocks density with connectivity between streets (rho = 0.75) and walkability (rho = 0.82); connectivity between streets with average size of the blocks (rho = -0.79) and walkability (rho = 0.87). Average size of the blocks with walkability (rho = -0.62). Existence of park and square with lower distance for park and square (rho = -0.82).

Middle (1km buffers) – High correlation between following variables: Land use mix with neighborhood recreation facilities (rho = 0.61). Residential density with connectivity between streets (rho = 0.76) and walkability (rho = 0.78). Connectivity between streets with blocks density (rho = 0.81), average size of the blocks (rho = -0.72) and walkability (rho = 0.93). Blocks density with walkability (rho = 0.89). Existence of bicycle path with lower distance for bicycle path (rho = -0.70).

High (0.5km buffers) – High correlation between following variables: Residential density with blocks density (rho = 0.66) and walkability (rho = 0.76); Blocks density with connectivity between streets (rho = 0.72) and walkability (rho = 0.82); connectivity between streets with average size of the blocks (rho = -0.75) and walkability (rho = 0.85). Existence of park and square with lower distance for park and square (rho = -0.79). Existence of bicycle path with lower distance for bicycle path (rho = -0.62).

High (1km buffers) – High correlation between following variables: Residential density with blocks density (rho = 0.75) and walkability (rho = 0.80). Connectivity between streets with blocks density (rho = 0.78), average size of the blocks (rho = -0.69) and walkability (rho = 0.86). Blocks density with walkability (rho = 0.87). Existence of bicycle path with lower distance for bicycle path (rho = -0.68).

Low (0.5km buffers) – High correlation between following variables: Residential density with blocks density (rho = 0.66) and walkability (rho = 0.79); Blocks density with connectivity between streets (rho = 0.84) and walkability (rho = 0.89). Connectivity between streets with average size of the blocks (rho = -0.69) and walkability (rho = 0.90); Existence of park and square with lower distance for park and square (rho = -0.86).

Low (1km buffers) – High correlation between following variables: Residential density with connectivity between streets (rho = 0.63), blocks density (rho = 0.77) and walkability (rho = 0.76). Connectivity between streets with blocks density (rho = 0.87), average size of the blocks (rho = -0.65) and walkability (rho = 0.91). Blocks density with walkability (rho = 0.88). Lower distance for bicycle path with existence of bicycle path (rho = -0.74).

Middle (0.5km buffers) – High correlation between following variables: Land use mix with neighborhood recreation facilities (rho = 0.66); residential density with blocks density (rho = 0.69) and walkability (rho = 0.78). Blocks density with connectivity between streets (rho = 0.78) and walkability (rho = 0.82); connectivity between streets with average size of the blocks (rho = -0.75) and walkability (rho = 0.88). Average size of the blocks with walkability (rho = -0.60). lower distance for park and square with existence of park and square (rho = -0.84) and blocks density (rho = 0.62).

Middle (1km buffers) – High correlation between following variables: Land use mix with neighborhood recreation facilities (rho = 0.66). Residential density with connectivity between streets (rho = 0.65), blocks density (rho = 0.80) and walkability (rho = 0.81). Connectivity between streets with blocks density (rho = 0.86), average size of the blocks (rho = -0.76) and walkability (rho = 0.90). Walkability with blocks density (rho = 0.90) and average size of the blocks (rho = -0.60). Existence of bicycle path with lower distance for bicycle path (rho = -0.68).

High (0.5km buffers) – High correlation between following variables: Land use mix with neighborhood recreation facilities (rho = 0.68). Residential density with blocks density (rho = 0.67) and walkability (rho = 0.78). Blocks density with connectivity between streets (rho = 0.70) and walkability (rho = 0.77). Connectivity between streets with average size of the blocks (rho = -0.76) and walkability (rho = 0.81). Existence of park and square with lower distance for park and square (rho = -0.82).

High (1km buffers) – High correlation between following variables: Land use mix with neighborhood recreation facilities (rho = 0.68); Residential density with blocks density (rho = 0.74) and walkability (rho = 0.73). Connectivity between streets with blocks density (rho = 0.73), average size of the blocks (rho = -0.75) and walkability (rho = 0.83). Blocks density with walkability (rho = 0.81). Existence of bicycle path with lower distance for bicycle path (rho = -0.64).
